# *Oviductus Ranae* Promotes Follicle Growth Through the PI3K/AKT Signaling Pathway In Vivo and In Vitro in Rat

**DOI:** 10.1002/fsn3.4621

**Published:** 2024-12-04

**Authors:** Hongyu Zhao, Yu Wang, Xueyuan Sun, Xingyao He, Lin Di, Zhen Yang

**Affiliations:** ^1^ Key Laboratory of TCM Pharmacology Jilin Academy of Chinese Medicine Sciences Changchun Jilin P.R. China; ^2^ College of Chemistry and Life Sciences Changchun University of Technology Changchun Jilin P.R. China

**Keywords:** follicles, ovary, *Oviductus Ranae*, PI3K/Akt

## Abstract

The development status of follicles determines the menstrual cycle and estrogen levels, which is crucial to women's health. *Oviductus Ranae* is a natural product for both medicine and food, which has “estrogenic effect”. However, few studies have systematically elaborated its mechanism of action. Hence, we hypothesize the “estrogen‐like effects” of OR may stem from its positive influence on the growth and development of growing follicles in the ovaries. In this study, the effect of *Oviductus Ranae* (OR) on the growth and development of rat follicles and follicles cultured in vitro was investigated. The content of estrogen in rat serum and follicular culture medium in vitro was determined by radioimmunoassay, and the levels of PI3K‐Akt signal pathway and FSHR expression in rat ovary and cultured follicles were detected by RT‐PCR and Western Blot. The follicles at different developmental stages in rat ovaries were analyzed by H&E and TUNEL staining. Follicles cultured in a medium containing OR displayed a significant increase in diameter, and the E2 content in the medium was significantly increased. Moreover, Rats treated with OR showed significant increases in estradiol and progesterone levels. The number of antral follicles in the ovaries displayed a significant increase, while the percentage of atretic antral follicles and the total atretic follicles showed significant decreases. The relative expression levels of PI3K, Akt, and FSHR were significantly increased, while significantly decreased in that of PTEN In Vivo and In Vitro. OR may promote follicle growth through PI3K/ Akt pathway, exhibiting “estrogen‐like effects”.

## Graphical abstract

Taking Oviductus Ranae can up‐regulate the PI3K/AKT Signaling Pathway in follicles, promote follicle growth in vivo and in vitro experiments, and these affected follicles will secrete more estrogen, which explains the "estrogen‐like effect" of Oviductus Ranae on the body.

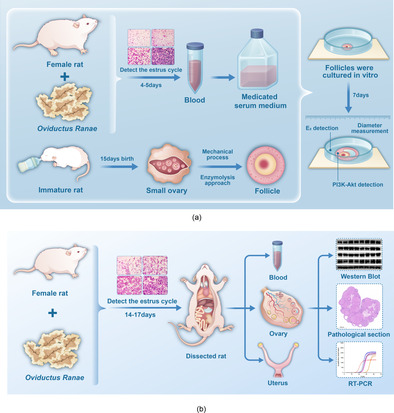

## 1 Background

Estrogens, quintessential for the maturation and preservation of female fecundity, encompass estradiol, estriol, and estrone, predominantly synthesized within ovarian follicles, with a supplementary secretion from the adrenals. The synthesis and systemic dissemination of estrogen are intricately modulated by the hypothalamic–pituitary–ovarian axis, with its systemic levels experiencing fluctuations in tandem with the ovarian follicular developmental cycle (menstrual cycle) (Grive and Freiman 2015). The advent of the perimenopausal epoch or the onset of premature ovarian senescence due to pathological conditions precipitates a notable diminution in follicular recruitment (McGee and Hsueh 2000; Zhang et al. 2014), or a hindrance in follicular maturation, culminating in a decline in estrogenic levels. This decrement instigates a spectrum of manifestations, including menstrual dysregulation, autonomic dysfunctions (Wu et al. 2005), and osteopenia (Watts 2018), and escalates the risk for cardiovascular and cerebrovascular pathologies (Mikkola and Clarkson 2002; Murphy, McCullough, and Smith 2004), thereby underscoring the imperative role of follicular maturation in safeguarding female health.

**FIGURE 1:**
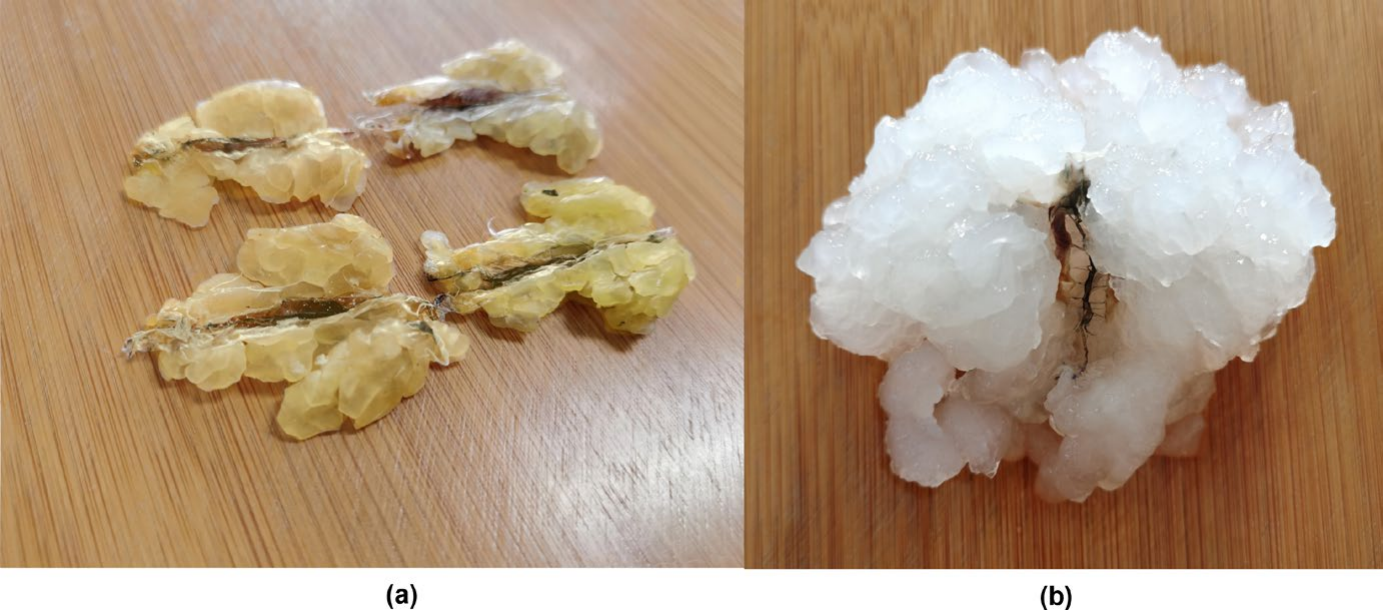
*Oviductus Ranae* (a) Dried *Oviductus Ranae* (b) *Oviductus Ranae* after soaking.

*Oviductus Ranae* (OR) (Figure 1), a traditional Chinese medicinal material derived from the desiccated oviducts of female *Rana temporaria* during the pre‐hibernation and post‐hibernation periods, revered as a dual‐purpose natural product for both medicinal and dietary uses, boasts centuries of application with an exemplary safety profile (Zhang et al. 2017). The main component of OR is protein, which accounts for more than 50%. It is also rich in steroid hormones, polysaccharides, phospholipids, fatty acids, and 1‐Methylhydantoin. (Tian, Sun, and Zhang 2023; Zhang et al. 2019; Wang et al. 2008). From the Ming Dynasty to the present in China, OR has been used as a classic nourishing ingredient for women. The ancients believed that OR could bring beauty and strong fertility to women. Modern research has found that taking OR can increase serum estrogen levels, thicken the uterine wall and endometrium, increase FSHR expression in the ovaries (Kang, Li, and Jiang 2015), and improve symptoms such as reproductive organ atrophy and osteoporosis caused by low estrogen levels in mice and rat. (Li et al. 2019; Wang et al. 2013; Liang et al. 2008). Clinical studies have shown that the use of OR can improve menopausal osteoporosis and increase serum estradiol levels in patients. (Wang, Ben, and Han 2014). Therefore, scientists generally believe that OR has “estrogen‐like effects”.

Given the rich steroid hormone content of OR, with estradiol and progesterone measured at approximately (52.3 ± 5.89) pg/100 g and (187.9 ± 19.4) pg/100 g, respectively (Zhang et al. 2019), scholars generally attribute its “estrogen‐like effects” to the exogenous intake of steroids such as estradiol. Preliminary research in our laboratory revealed that oral administration of OR elevated serum estradiol levels and thickened the endometrial lining in female rats. In ovariectomized rats, endometrial thickness increased without significantly affecting serum estradiol levels. After 8 weeks of administration (equivalent to 11–14 estrous cycles in rats), serum estradiol levels actually decreased, and the presence of numerous degenerated structures, originating from former corpora lutea or interstitial glands in the ovaries, akin to a short‐term decline following ovarian hyperstimulation (Zhao et al. 2019, 2021), indicating that OR's “estrogen‐like effects” do not align with the characteristics of exogenous estrogen supplementation.

In our previous research, we found that differential gene expression in the ovarian transcriptome of rats short‐term fed with OR was primarily enriched in the phosphatidylinositol 3 kinase (PI3K) /protein kinase B(Akt) signaling pathway (He et al. 2023), essential for follicular growth and development. This pathway plays a crucial role in ovarian functions such as the recruitment of primordial follicles, proliferation of granulosa cells, corpus luteum formation, and oocyte maturation (Adhikari and Liu 2009; Makker, Goel, and Mahdi 2014). Upregulation of the PI3K/Akt signaling pathway in the ovary may affect the development of ovarian follicles. Hence, we hypothesize the “estrogen‐like effects” of OR may stem from its positive influence on the growth and development of growing follicles in the ovaries, leading to the design of this experiment to explore its impacts on both in vivo and in vitro follicular development.

## 2 Methods

### 2.1 Preparation of *Oviductus Ranae*

OR, provided by Tonghua Dezhen Tang Wild Resources Development Co. Ltd., was authenticated by Director Pharmacist Di Lin from Jilin Academy of Chinese Medicine Sciences as the dried oviduct product of the female *Rana temporaria chensinensis* David. The preparation followed traditional methods, pre‐soaking in distilled water at a ratio of 1:100 for 16 h to form a characteristic jelly‐like gel (Figure 1b). This gel, after thorough cutting and stirring with a high‐speed mixer, achieved a uniform and moderately viscous pseudoplastic fluid, ready for oral administration to rats.

### 2.2 Animal and Ethical Considerations

All experiments were approved by the Experimental Animal Ethics Committee of the Jilin Provincial Academy of Traditional Chinese Medicine, in accordance with the committee's regulations (Approval No. JLSZKYDWLL‐2021‐019). Seventy female WISTAR rats (8 weeks old, weight 200 ± 20 g) and five male WISTAR rats (8 weeks old, weight 300 ± 20 g), supplied by Liaoning Changsheng Biotechnology Co. Ltd., were housed in the barrier facility IVC cages of Jilin Academy of Chinese Medicine Sciences. The facility maintained constant temperature, humidity, and light cycles (12 h light/12 h dark), with animals having free access to food and water.

### 2.3 In Vitro Experiment Preparation of Medicinal Serum and Culture Medium

**FIGURE 2:**
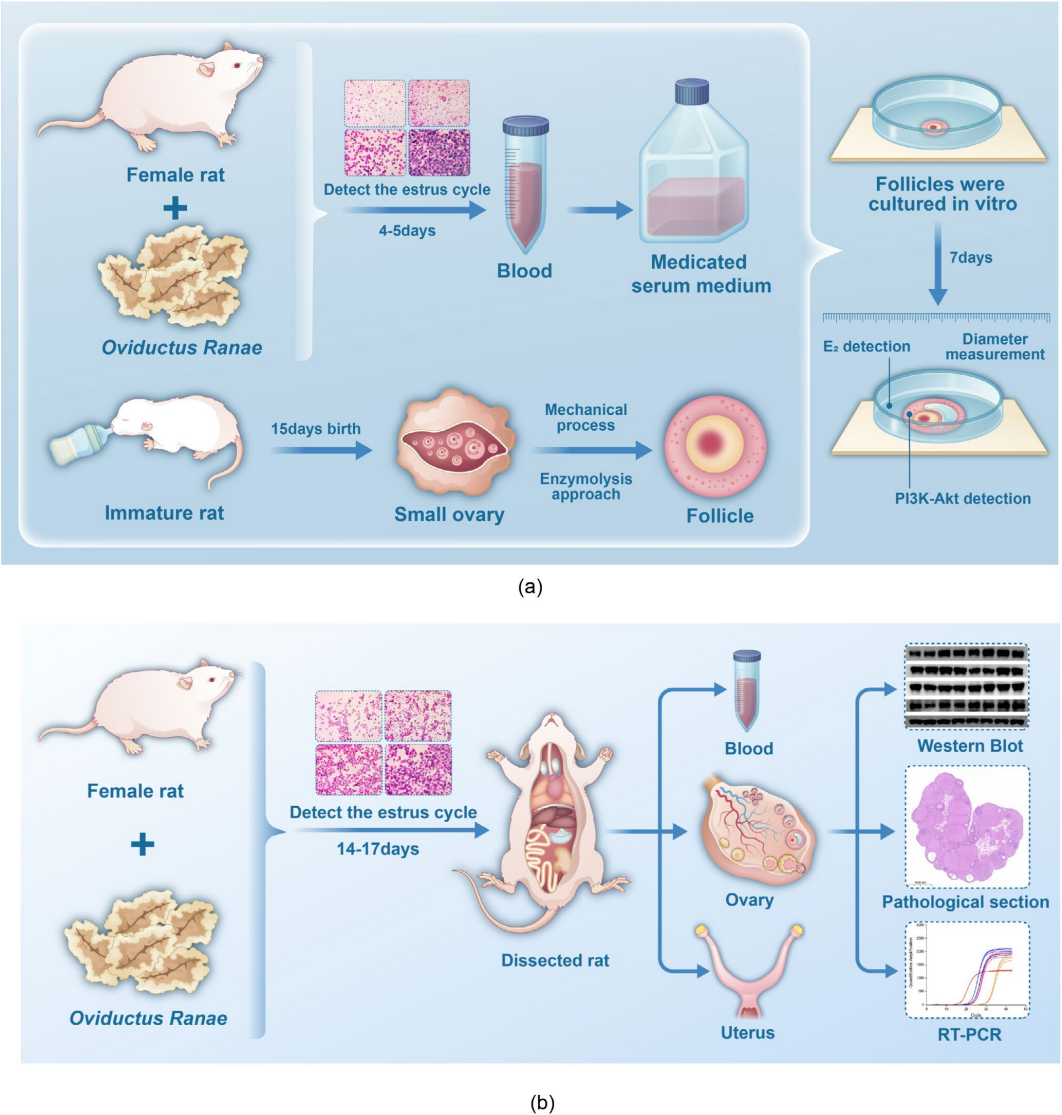
Experimental protocol flow (a) Experimental procedure of *Oviductus Ranae* on the growth of Rat follicles in vitro. Female rats during estrus were gavage with *Oviductus Ranae* for 4–5 days. When proestrus was reached again, blood was taken to prepare *Oviductus Ranae* drug‐containing serum medium; the ovaries of 15‐day‐old female rat were taken, and follicles were isolated by enzymatic + mechanical methods and cultured in *Oviductus Ranae* drug‐containing medium to measure changes in diameter, estradiol secretion, and mRNA level of PI3K‐Akt signaling pathway in follicles during growth. (b) Experimental procedure of *Oviductus Ranae* on the growth of Rat follicles in vivo. Female rats were gavage with *Oviductus Ranae* or other drugs for 14–17 days. When they reached proestrus, they were sacrificed to collect blood, ovary, uterus, and other samples to determine serum sex hormone content, uterine weight index, ovarian weight index, histopathological changes, changes in the number of follicles at different development stages, and the mRNA and protein levels of the PI3K‐Akt signaling pathway.

After 1 week of acclimatization to the facility, nine estrous cycle‐normal female WISTAR rats in the inter‐estrus phase were selected and divided into three groups: control, high‐dose OR, and low‐dose OR (*n* = 3). The high‐ and low‐dose groups received 400 and 200 mg/kg of OR via oral gavage, respectively, in a volume of 20 mL/kg, while the control group received an equivalent volume of distilled water. Following this regimen, the estrous cycle was monitored daily. At the pre‐estrus phase (about 4–5 days later), rats were anesthetized with 40 mg/kg pentobarbital sodium for blood collection via the abdominal aorta. The blood was centrifuged at 3000 rpm to separate the serum, which was then inactivated at 56°C and filtered through a 0.22 μm membrane to produce medicinal serum. This serum was used to prepare the culture medium containing 10% rat serum, 5% fetal bovine serum, 100 mU/mL FSH, 1% ITS‐mix, 100 IU/mL penicillin, 0.1 mg/mL streptomycin, and 10 mmol/L HEPES buffer in α‐MEM, adjusted to pH 7.2–7.3. (Figure 2a).

### 2.4 Preantral Follicle Isolation, Culture, and Assessment

Ten female WISTAR rats and five male WISTAR rats were co‐housed in a 2:1 ratio for mating, with offspring at 15 days post‐birth utilized for preantral follicle isolation. The juvenile rats were euthanized using a CO_2_ chamber, followed by a 5‐min immersion in 75% ethanol before transfer to a sterile workbench. Under aseptic conditions, bilateral ovaries were excised, placed in washing medium (containing 10% fetal bovine serum, 100 IU/mL penicillin, and 0.1 mg/mL streptomycin in L15 medium), cleansed of connective tissue, and sectioned into 1 mm^3^ pieces before enzymatic digestion in enzymatic solution (comprising 10% fetal bovine serum, 2 mg/mL Type I collagenase, 0.2 mg/mL DNase I, 100 IU/mL penicillin, and 0.1 mg/mL streptomycin in L15 medium) at 37°C for 20 min. The digest was then filtered through a 100 μm cell strainer, thoroughly washed to remove enzymes, and the follicles transferred to a 96‐well plate containing standard follicle culture medium (with 10% fetal bovine serum, 100 mU/mL FSH, 1% ITS‐mix, 100 IU/mL penicillin, 0.1 mg/mL streptomycin, and 10 mmol/L HEPES buffer in α‐MEM medium) (Wan et al. 2011; Figueiredo et al. 1993). Each well received one follicle, and under an inverted microscope (Leica DFC295), follicles approximating spherical shape, 110–130 μm in diameter, with intact basement membrane and granulosa cell layers were selected and randomly assigned to four groups: blank control (BC group), rat serum control (RSC group), high‐dose OR(ORH group), and low‐dose OR(ORL group) (*n* = 100), and cultured in a CO_2_ incubator (SANYO Electric Co. Ltd. MCO‐18AIC(UV)) at 37°C, 5% CO_2_. On Day 2, following adhesion and growth, the culture medium for the RSC, ORH, and ORL groups was switched to their corresponding medicated rat serum medium, while the BC group follicles also underwent medium change, continuing with the standard follicle culture medium. All cultured follicles had their medium semi‐quantitatively changed every other day, with the spent medium collected and stored at −80°C, follicle growth documented photographically, and follicle diameters measured using ImageJ software, over a continuous 7‐day culture. Throughout the culturing process, a fraction of the follicles in all groups ceased growth or exhibited oocyte release due to basement membrane rupture. Thus, the final statistics for follicle diameter and estradiol concentration in the culture medium were based on follicles that continued to grow and maintained a generally normal appearance over 7 days of culture. Fifty follicles were selected from each group, and the culture mediums collected from these follicles were pooled by group and day of culture in quintuples for estradiol concentration determination.

On the final day, after photographing and collecting the spent culture medium, adherent follicles were scraped off, grouped by treatment, centrifugally concentrated together, and preserved in liquid nitrogen. (Figure 2a).

### 2.5 In Vivo Experiments Animal Grouping and Treatment

After a week of acclimatization to the facility, 32 rats with regular estrous cycles were selected and randomly divided into four groups: a blank control (BC) group, a positive control (PC) group, along with high‐dose (ORH) and low‐dose (ORL) OR groups (*n* = 8 each). The positive control group received oral administration of 1 mg/kg diethylstilbestrol and 0.8 mg/kg medroxyprogesterone acetate, while the high‐ and low‐dose groups were administered OR at 400 and 200 mg/kg, respectively, doses derived from human clinical doses adjusted for rat equivalent doses (Nair and Jacob 2016). Administration continued for 14–17 days (as euthanasia for sample collection required the animals to be in the proestrus stage, the number of dosing days varied). On the first day of proestrus following 14 days of administration, rats were anesthetized with sodium pentobarbital, blood was collected from the abdominal aorta for serum separation, and both ovaries were extracted for histopathological and molecular examinations. (Figure 2b).

### 2.6 Estrous Cycle Detection

Vaginal secretions were collected by saline lavage for rapid Gram staining, and the type of epithelial cells present was observed under a light microscope to determine the stage of the estrous cycle (Ndeingang et al. 2019). Females displaying a regular sequence of proestrus, estrus, metestrus, and diestrus over 4–5 days were defined as having a normal estrous cycle. The interval between the first and second proestrus stages detected after 10 days of medication was recorded as the length of the estrous cycle.

### 2.7 Serum and Culture Medium Hormone Determination

The contents of estradiol (E_2_), progesterone (P), testosterone (T), follicle‐stimulating hormone (FSH), and luteinizing hormone (LH) in rat serum, as well as the concentration of E_2_ in the in vitro follicle culture medium, were all measured using competitive radioimmunoassay kits (Beijing North Institute of Biological Technology, Beijing, China).

### 2.8 Ovarian H&E Staining, TUNEL Staining, and Counting of Follicles at Different Developmental Stages

The left ovary, once excised and washed with saline, was fixed in 10% formalin for 48 h, dehydrated with graded alcohol for 8 h, transparentized with graded xylene for 4 h, and immersed in graded paraffin for 6 h. Finally, the ovary was embedded in melted paraffin. Sections of 5 μm thickness at the largest cross‐sectional area of the ovary were prepared for consecutive H&E and TUNEL staining, followed by whole‐slide scanning. Follicles in preantral stages (primary and secondary follicles), antral follicles, and corpora lutea were counted in H&E‐stained sections, while follicular atresia were assessed and counted in TUNEL‐stained sections. The atresia rates for preantral follicles, antral follicles, and the total follicular population were calculated separately, excluding primordial follicles from the analysis (Table 1).

**TABLE 1:** Primers used for qRT‐PCR.

Gene symbol	Sequence (5′‐3′)	Product length (bp)
PTEN	(F) TGTAAAGCTGGAAAGGGACG (R) CCTCTGACTGGGAATTGTGAC	20 21
PI3K	(F) GGATGCTGAATGGTACTGGG (R) TGTAAGAGTGTAATCGCCGTG	20 21
Akt	(F) GCCCTCAAGTACTCATTCCAG (R) ACACAATCTCCGCACCATAG	21 20
FSHR	(F) TACAGCTCTGCCATGCTGCC (R) GCGTTGAGTACGAGGAGGGC	20 21
*β*‐actin	(F) ACCTTCTACAATGAGCTGCG (R) CTGGATGGCTACGTACATGG	20 20

### 2.9 mRNA Isolation and RT‐PCR

Total RNA was extracted from the right ovaries of each group of rats and from the 7‐day cultured follicles using TRIzol reagent (Invitrogen, USA). The RNA was reverse transcribed into cDNA using the ABScript III RT Master Mix for qPCR with gDNA Remover kit (ABclonal Technology, Wuhan, China). Real‐time PCR was conducted using BioEasy Master Mix (SYBR Green, Low ROX) fluorescent dye (BioFlux, Beijing, China) and the Agilent Stratagene Mx3000P real‐time fluorescent quantitative PCR instrument. To conduct the reaction program, we initiated a denaturation process at 95°C for 30 s. This was followed by 40 cycles of denaturation at 95°C for 10 s, annealing at 57°C for 30 s, and extension at 72°C for 30 s. **Table** contains a list of the primers used in the process. To ensure accuracy, we utilized the β‐actin value as an internal reference and employed the 2^−ΔΔCt^ method for relative quantification analysis.

### 2.10 Protein Extraction and Western Blot

The right ovaries of each group of rats were ground using a tissue grinder (Gering Scientific Instruments, Beijing, China), and the proteins were extracted by incubating the samples in RIPA buffer (Beyotime Biotechnology, Shanghai, China) at low temperature for 30 min, followed by centrifugation. The protein concentration in the supernatant was determined using a BCA assay kit (Beyotime Biotechnology, Shanghai, China). The extracted proteins were separated by SDS‐PAGE (Beijing Dingguo Changsheng Biotechnology, Beijing, China) and transferred to PVDF membranes (Millipore, USA). The membranes were blocked with 5% BSA in TBST buffer for 20 min, followed by incubation with primary antibodies against PI3K (PI3 Kinase p110 delta Rabbit mAb, ABclonal Technology, Wuhan, China), Akt (AKT1 Rabbit mAb, ABclonal Technology, Wuhan, China), phosphorylated Akt (p‐Akt) (Phospho‐Akt‐S473 Rabbit mAb, ABclonal Technology, Wuhan, China), FSH receptor (FSHR) (FSHR Rabbit pAb, ABclonal Technology, Wuhan, China), and β‐actin (β‐Actin Rabbit mAb, ABclonal Technology, Wuhan, China) diluted in TBST buffer (1:1000) at room temperature for 20 min. After washing with TBST buffer, the membranes were treated with diluted secondary antibodies in TBST buffer (1:2000) and left at room temperature for 20 min. After washing with TBST buffer, the PVDF membranes were immersed in TBST solution, and the chemiluminescent substrate ECL (Millipore, USA) was added to the target protein bands. After incubating the membranes in darkness at room temperature for 2 min, the protein bands were made visible through a chemiluminescence imaging system (Tanon 4600). The grayscale values of the bands were then analyzed using Image J software in a quantitative manner.

### 2.11 Statistics

Quantitative data were analyzed using GraphPad Prism 9. For comparisons between multiple sample means, the one‐way ANOVA test was applied for data with normal distribution and homogeneity of variance, with pairwise comparisons conducted using Dunnett's test; the Welch test for data with unequal variances, with pairwise comparisons using Dunnett's T3 method; and for non‐normally distributed data, the Kruskal–Wallis *H* test (rank sum test) with pairwise comparisons using Dunnett's method. Quantitative data are expressed as mean ± standard deviation, with *p* < 0.05 indicating statistical significance.

## 3 Results

### 3.1 In Vitro Follicle Growth Status, Diameter, and E_2_ Concentration in Culture Medium

**FIGURE 3:**
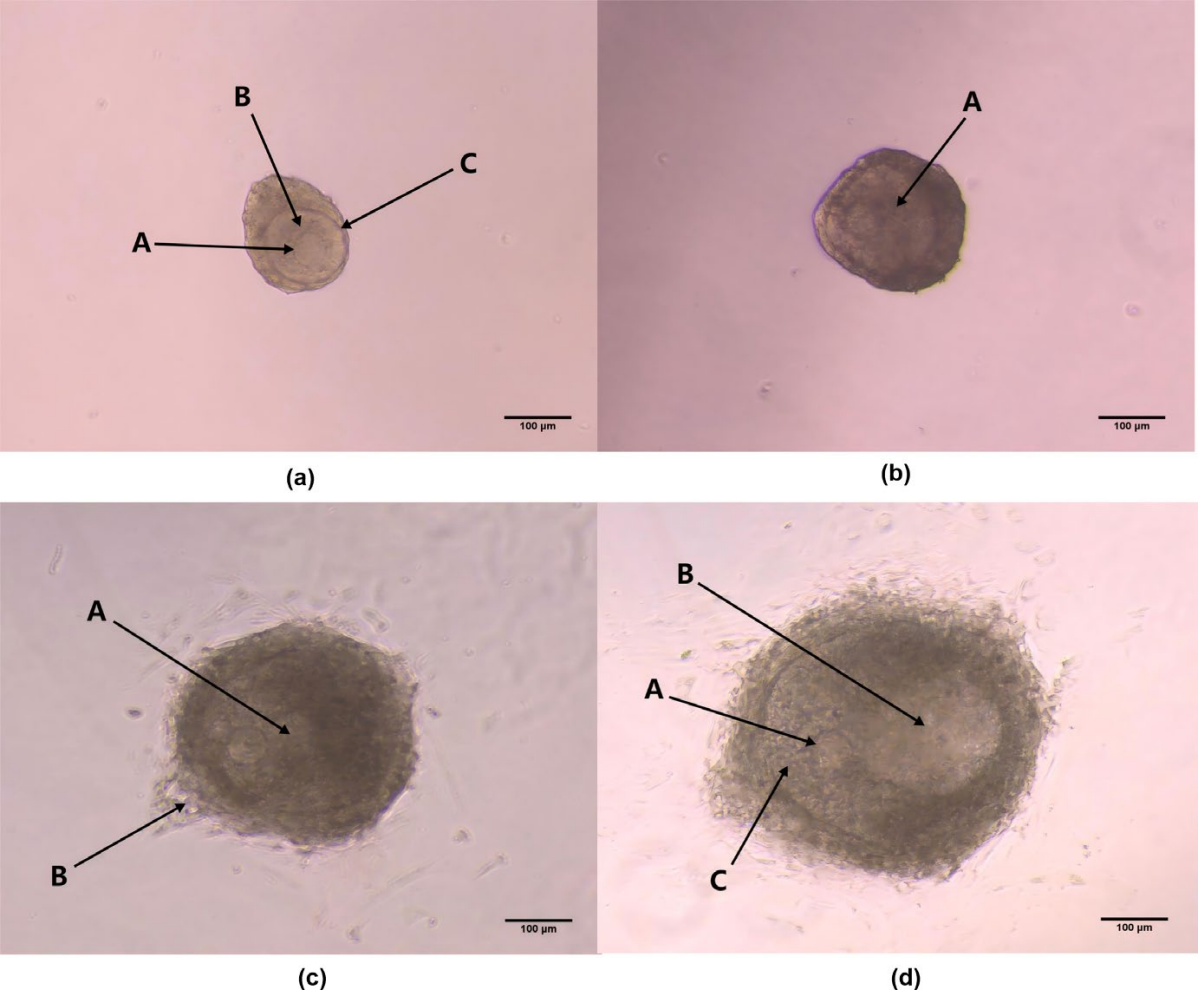
Growth status of follicles in vitro. (a) Follicle just separated from the ovary, the arrow A points to the oocyte, the arrow B points to the granulosa cell, and the arrow C points to the theca cell, scale bar = 100 μm. (b) Follicle cultured in vitro for 1 day, began to grow attached to the wall, arrow A points to the proliferating granulosa cell layer, scale bar = 100 μm. (c) Follicle cultured in vitro for 3 days. Arrow A points to the newly developed sinus structure, and arrow B points to the phenomenon of granulosa cells breaking through the basement membrane, scale bar = 100 μm. (d) Follicle cultured in vitro for 7 days. The follicle develops to the stage of mature follicle. Arrow A points to the oocyte near the edge of the follicle, arrow B points to the huge antrum filled with follicular fluid, and arrow C points to the cumulus structure, scale bar = 100 μm.

Fifteen days post‐birth of rats, preantral follicles isolated from ovaries exhibited near‐spherical shapes, with relatively thin granulosa cell layers and visibly distinct oocytes (Figure 3a). After 1 day of culture, follicles settled at the bottom of the medium, adhering to the surface and their originally spherical structure began to collapse, presenting a “fried egg” morphology (Figure 3b). Rapid growth was observed within the medium, with some follicles developing antral cavities and others exhibiting granulosa cell proliferation beyond the basement membrane by Day 3 (Figure 3c). By Day 5, both follicle diameter and antral volume continued to expand. By Day 7, a portion of the follicles developed cumulus structures, indicating preliminary mature follicle morphology (Figure 3d).

**FIGURE 4:**
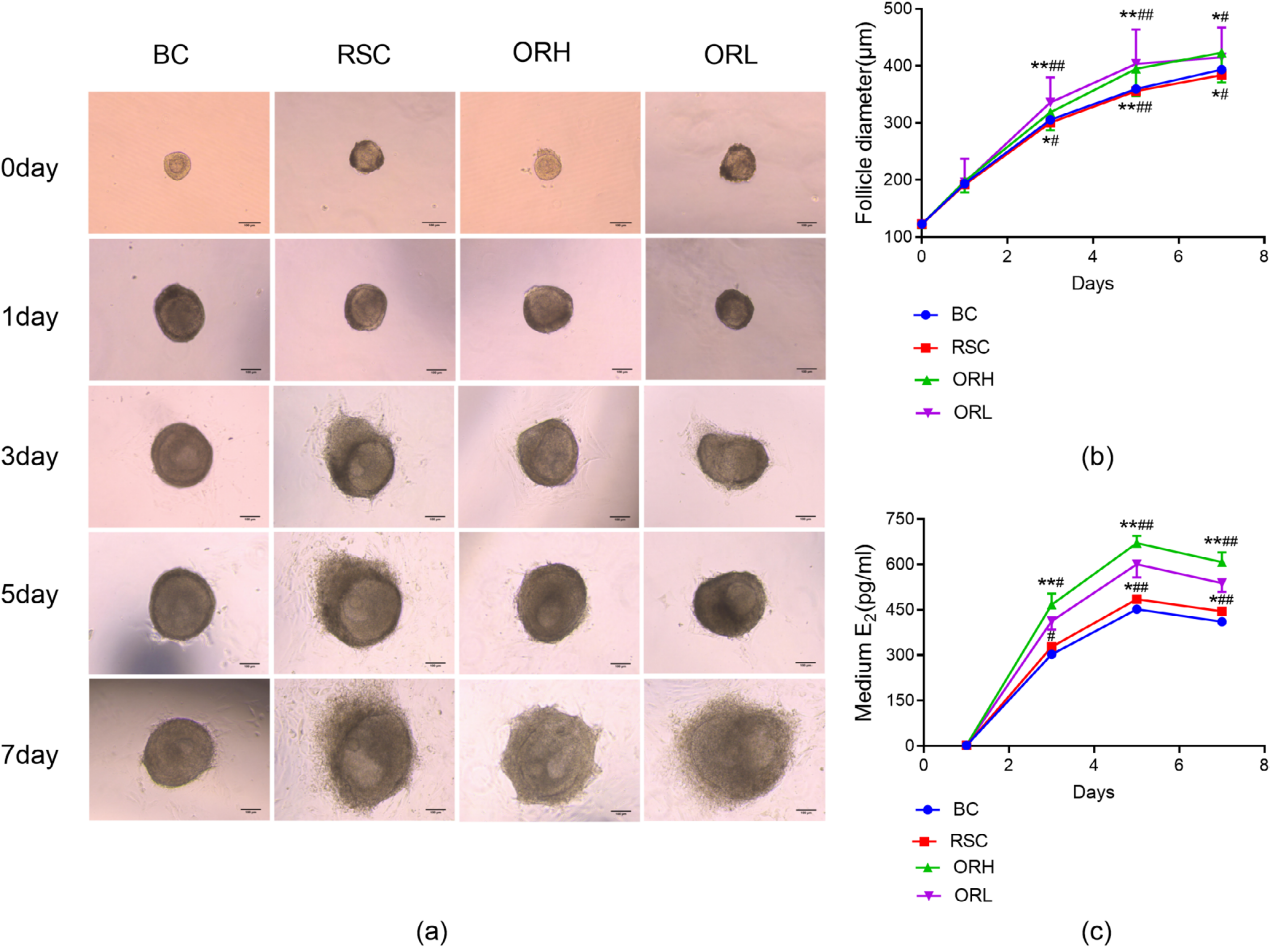
Effect of OR on follicle growth and estradiol secretion in vitro. (a) Representative images of in vitro grown follicles from each group at 0, 1, Days 3, 5, and 7 of culture, scale bar = 100 μm. (b) Changes in follicle diameter during Days 0–7 of in vitro culture for each group, with 50 follicle samples per group. (c) Changes in E_2_ concentration in the culture medium of follicles during Days 0–7 of in vitro culture for each group, with 10 culture medium samples per group. BC, blank control group; ORH, high‐dose OR group; ORL, low‐dose OR group; RSC, rat serum control group. Data are represented as the mean ± SD. *p < 0.05 and **p < 0.01 versus BC group; # *p* < 0.05; ^##^ *p* < 0.01 versus RSC group.

The diameter of follicles in all groups increased steadily over 7 days of culture. Due to flattening upon adhesion, the diameter increase was more pronounced from 0 to 1 day. From Days 1 to 7, the increase in follicle diameter was relatively stable, with the ORH and ORL groups showing a faster rate of increase from Days 1–5, which slightly decreased from Days 5–7. Compared to the BC group, the follicle diameters in the RSC group showed no significant differences at Days 1, 3, 5, and 7 (*p* > 0.05); the ORH group's follicles were significantly larger than those of the RSC group at Days 3 and 7 (*p* < 0.05), and markedly larger at Day 5 (*p* < 0.01); the follicles of the ORL group were significantly larger than those of the RSC group at Days 3 and 5 (*p* < 0.01), and noticeably larger at Day 7 (*p* < 0.05). (Figure 4a,b).

Throughout the incubation period of 1–5 days, the escalation in E_2_ concentration within the follicle culture mediums was pronounced, followed by a universal decrement from Days 5 to 7 across all groups, signifying a similar trajectory of E_2_ concentration changes. This phenomenon is attributed to the follicles nearing their growth zenith around Days 5–6, and the absence of LH in the medium, preventing ovulation and thus leading to a diminution in both the quality and activity of granulosa cells, resulting in a reduction of E_2_ secretion. Compared with the BC group, the E_2_ concentration in the medium of RSC group exhibited no significant variance on Days 3, 5, and 7 (*p* > 0.05); however, the medium of ORH group demonstrated a notable elevation in E_2_ levels on Days 5 and 7 compared to the RSC group (*p* < 0.05); the ORL group's medium showed a significant augmentation in E_2_ concentration on Days 3, 5, and 7 in comparison with the RSC group (*p* < 0.01). (Figure 4c).

### 3.2 Expression of PI3K, Akt, PTEN, and FSHR mRNA in Follicles In Vitro

**FIGURE 5:**
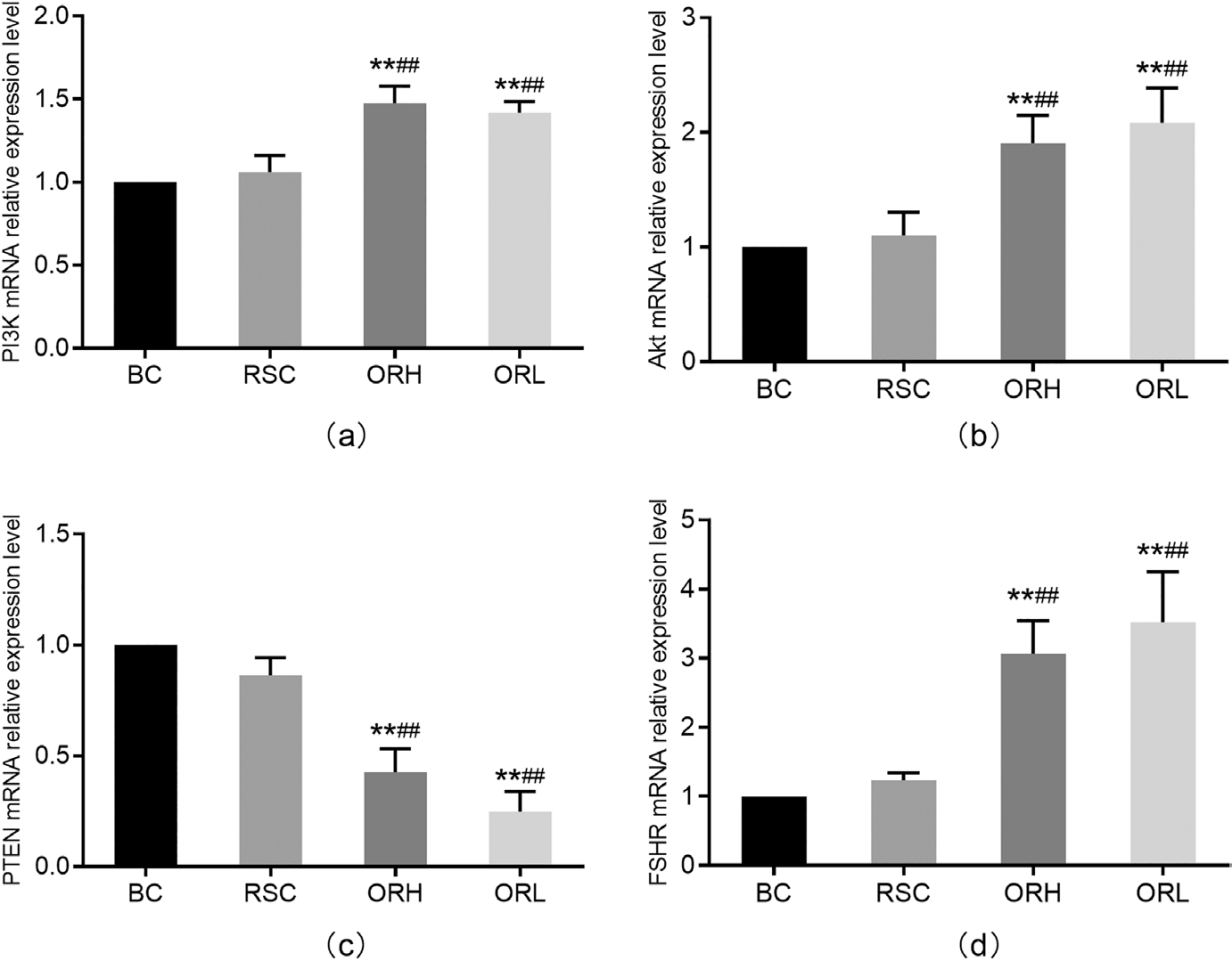
Effects of OR on mRNA expression of PI3K, Akt, FSHR, and PTEN mRNA in follicles in vitro. (a) showed the relative expression levels of PI3K mRNA in in vitro cultured follicles for 7 days relative to β‐Actin mRNA. (b) showed the relative expression levels of Akt mRNA in in vitro cultured follicles for 7 days relative to β‐Actin mRNA. (c) showed the relative expression levels of FSHR mRNA in in vitro cultured follicles for 7 days relative to β‐Actin mRNA. (d) showed the relative expression levels of PTEN mRNA in in vitro cultured follicles for 7 days relative to β‐Actin mRNA. The data from each group were normalized based on the BC group. BC, blank control group; ORH, high‐dose OR group; ORL, low‐dose OR group; RSC, rat serum control group. Data are represented as the mean ± SD with 3 batches of follicle samples. *p < 0.05 and **p < 0.01 versus BC group; ^#^ *p* < 0.05; ^##^ *p* < 0.01 versus RSC group.

No significant differences were found in the relative expression levels of PI3K, Akt, PTEN, and FSHR mRNA when comparing the RSC group to the BC group (*p* > 0.05). However, a significant increase in the relative expression levels of PI3K, Akt, and FSHR mRNA (*p* < 0.01) was observed when comparing the RSC group to the ORH and ORL groups (Figure 5a,b,d). Additionally, the relative expression level of PTEN mRNA significantly decreased (*p* < 0.01) (Figure 5c).

### 3.3 Rat Body Weight and Ovarian/Uterine Mass

**FIGURE 6:**
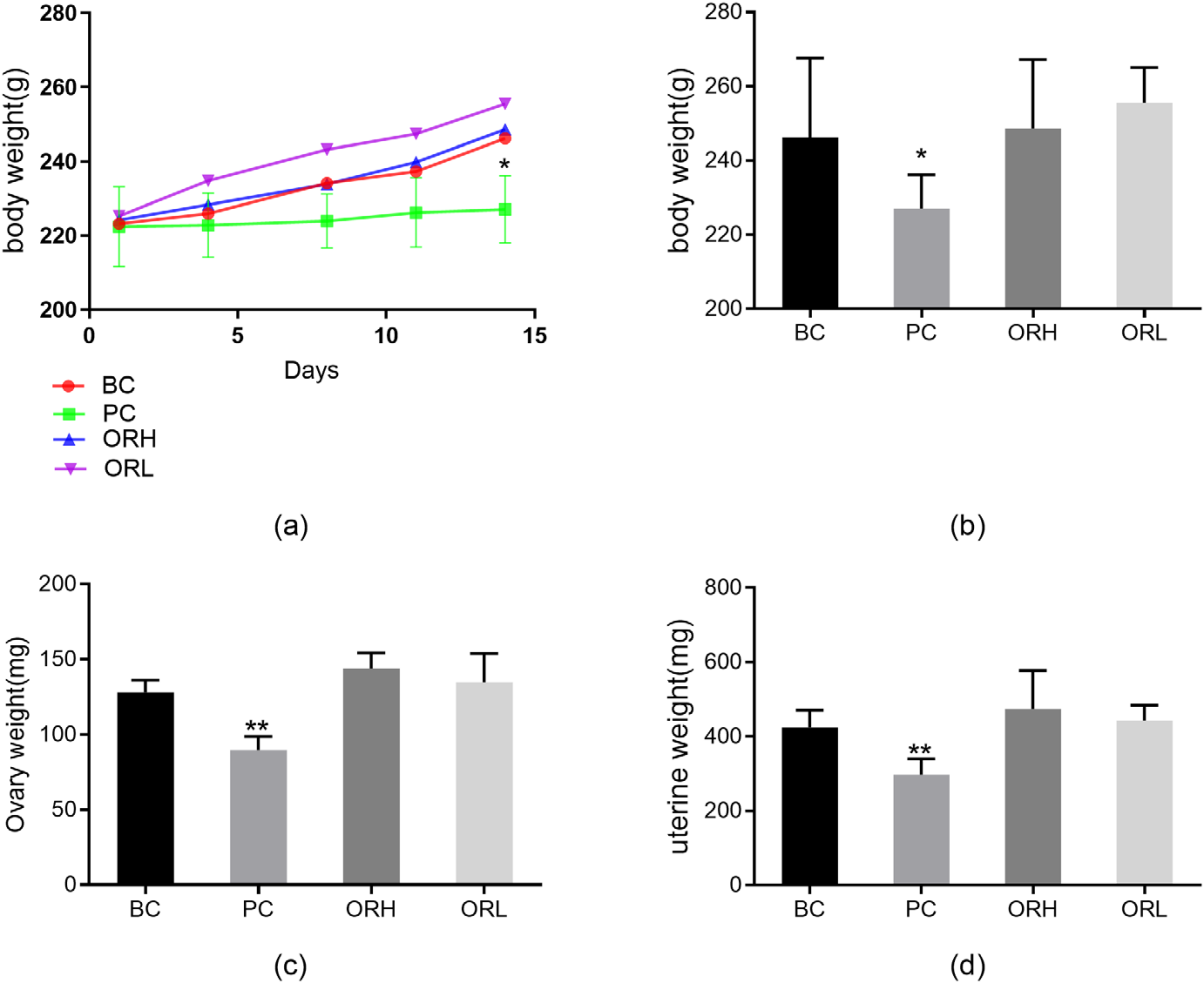
Effects of OR on bodyweight, ovary weight and uterine weight of rats. (a) Changes in body weight of rats in each group from Day 1 to 14 of gavage. (b) Final body weight of rats in each group. (c) Ovarian weight of rats in each group. (d) Uterine weight of rats in each group. BC, blank control group; ORH, high‐dose OR group; ORL, low‐dose OR group; PC, positive control group. Data are represented as the mean ± SD from 8 animals in each group. *p < 0.05 and **p < 0.01 versus BC group.

Regarding corporeal mass, there was no significant initial disparity among the groups. In comparison with the BC group, the PC group experienced a deceleration in weight gain (Figure 6a), and by Day 14, the PC group's weight conspicuously diminished relative to the BC group (*p* < 0.05), whereas no significant differences were observed between the ORH and ORL groups and the BC group (*p* > 0.05) (Figure 6b).

Contrastingly, when compared to the BC group, both ovarian and uterine weights in the PC group were significantly reduced (*p* < 0.01), a phenomenon considered to be associated with the inhibition of uterine muscular and endometrial thickening by medroxyprogesterone acetate; no notable changes were observed in the ovarian and uterine weights of the ORH and ORL groups in comparison with the BC group (*p* > 0.05). (Figure 6c,d).

### 3.4 Rat Estrous Cycle and Serum Hormonal Content

All rats exhibited a normative estrous cyclicity. In the proestrus phase, the PC group demonstrated augmented vaginal secretion and cellular collection, yet the estrous cycle remained unaltered. Serum utilized for hormonal assays across all groups was procured during the proestrus phase.

**FIGURE 7:**
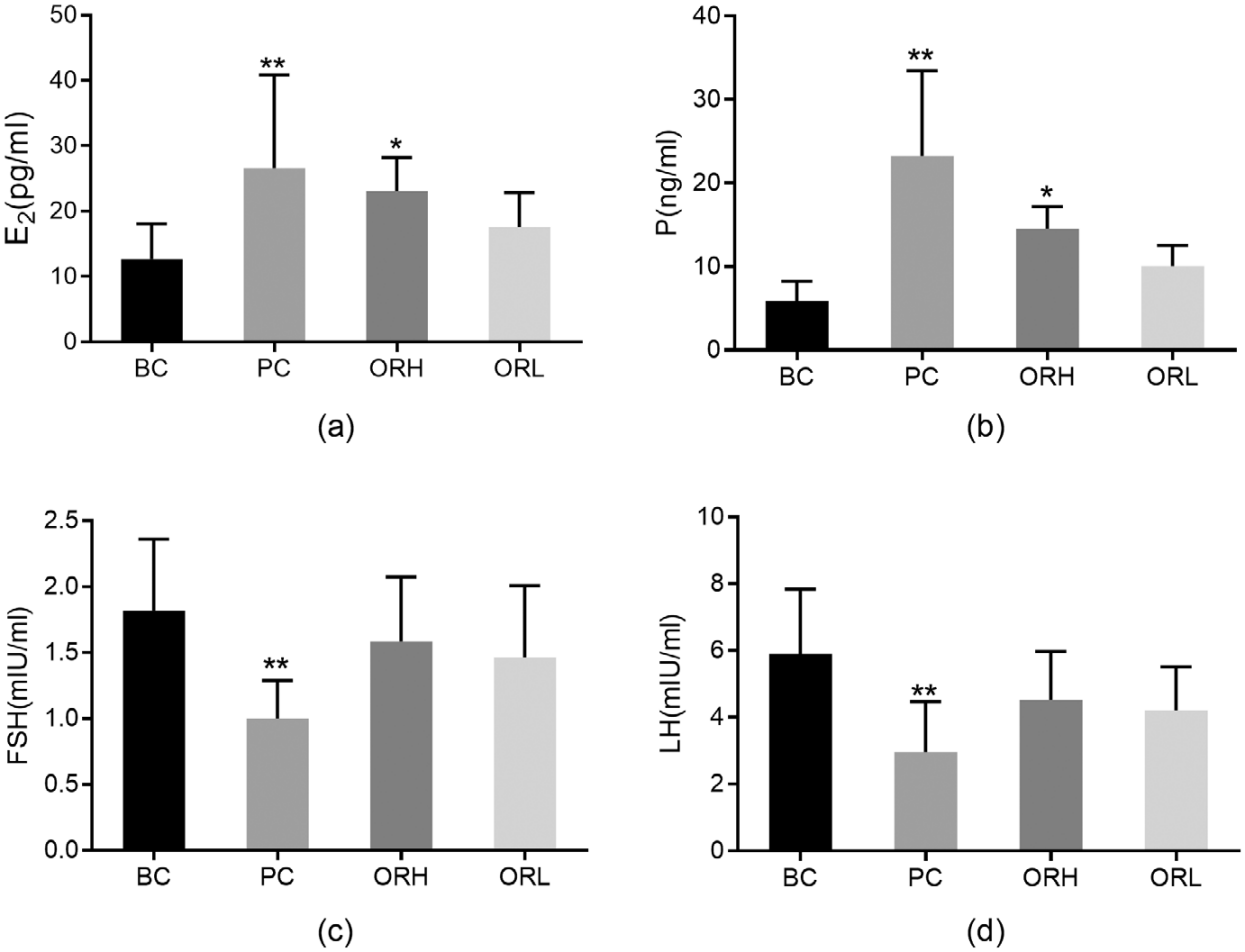
Effects of OR on sex hormone in serum of rats. (a) E_2_ levels. (b) P levels. (c) FSH levels. (d) LH levels. BC, blank control group; ORH, high‐dose OR group; ORL, low‐dose OR group; PC, positive control group. Data are represented as the mean ± SD from 8 animals in each group. *p < 0.05 and **p < 0.01 versus BC group.

Compared to the BC group, serum E_2_ and P levels in the PC group significantly escalated (*p* < 0.01), with FSH and LH markedly reduced (*p* < 0.01), aligning with exogenous hormone administration manifestations; the ORH group showed notable increases in serum E_2_ and P (*p* < 0.05), with FSH and LH levels remaining stable (*p* > 0.05). (Figure 7).

### 3.5 Rat Ovarian Follicle Count at Various Developmental Stages

**FIGURE 8:**
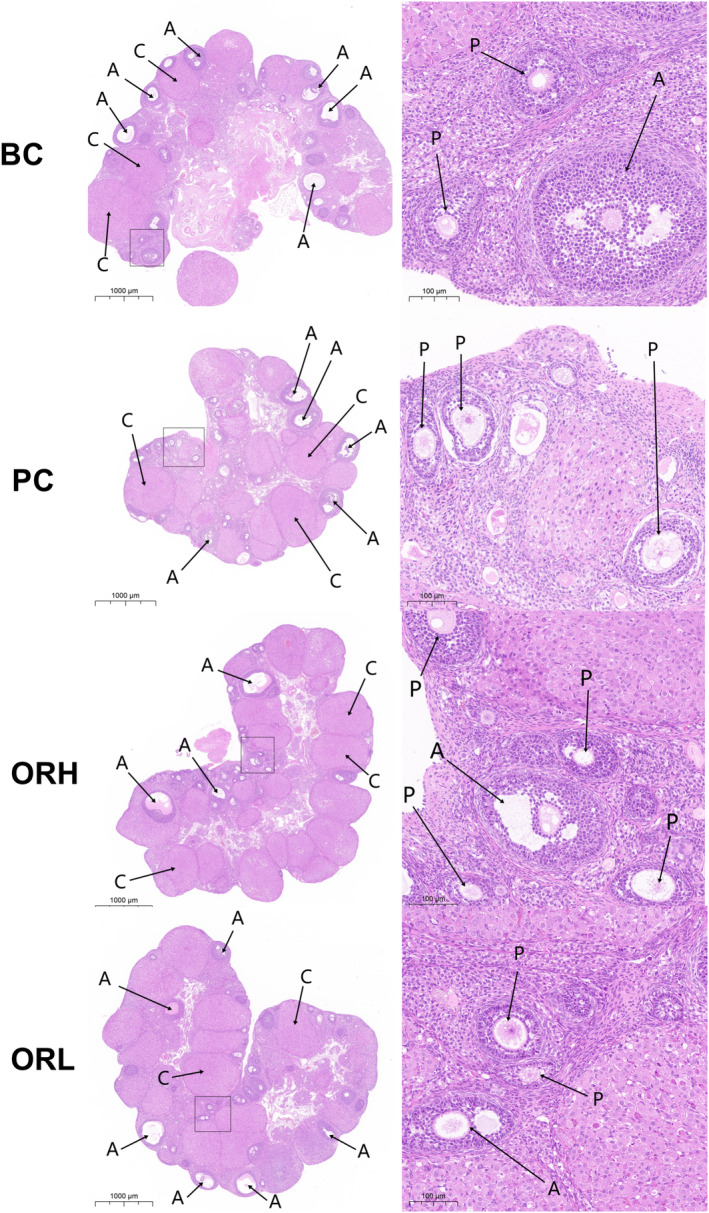
H&E staining of ovarian. Consecutive sections of the ovaries stained with H&E, allowing observation of follicles at different developmental stages. The high‐power image of ovary is an enlargement of the insert in the low‐power image, with scale bars of 100 and 1000 μm, respectively. The C arrows indicate corpora lutea, the A arrows indicate antral follicles, and the P arrows indicate preantral follicles.

**FIGURE 9:**
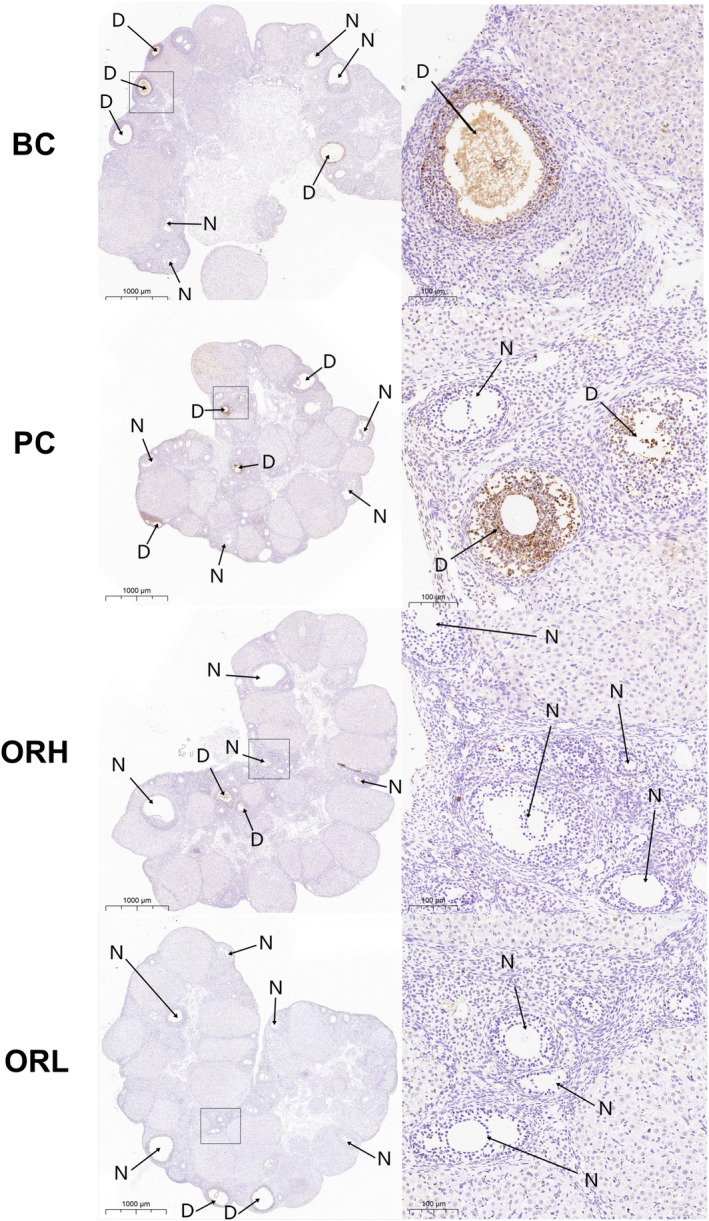
TUNEL staining of ovarian. Consecutive sections of the ovaries stained with TUNEL to observe the development and atresia of follicles. The high‐power image of ovary is an enlargement of the insert in the low‐power image, with scale bars of 100 and 1000 μm, respectively. The D arrows indicate follicles at various stages of atresia. The N arrows point to normally developing follicles.

H&E‐stained ovarian sections revealed follicles at diverse developmental stages and fresh corpora lutea within the cortex, supported by stromal connective tissue, with the medulla showcasing abundant vasculature (Figure 8). TUNEL‐stained sections distinctly identified follicles at various stages undergoing atresia (positive apoptotic granulosa cells) (Figure 9).

Relative to the BC group, the ovarian sections of PC group were generally smaller, with no significant difference in the number or atresia rate of preantral and antral follicles (*p* > 0.05); the ORH group exhibited a significant increase in antral follicle numbers (*p* < 0.05) and a substantial decrease in atresia rates (*p* < 0.01), with overall follicular atresia rates significantly reduced (*p* < 0.05); the ORL group showed a marked decrease in antral follicle atresia rates (*p* < 0.05), with no significant difference in corpora lutea numbers across groups. (Figures 8 and 9, Table 2).

**TABLE 2:** Effects of OR on follicles at various stages of development in ovary of rats.

Group	Preantral follicle (EA)	Antral follicle (EA)	Corpora lutea (EA)	Atresia rate of preantral follicle (%)	Atresia rate of antral follicle (%)	Atresia rate of follicle (%)
BC	9.88 ± 4.36	11.50 ± 2.78	9.00 ± 2.45	16.54 ± 5.83	47.39 ± 10.84	33.55 ± 9.16
PC	7.62 ± 1.77	10.63 ± 1.92	8.62 ± 21.68	18.40 ± 11.13	54.98 ± 8.63	39.86 ± 8.25
ORH	13.88 ± 4.16	14.63 ± 2.13*	10.88 ± 2.03	13.00 ± 3.42	31.88 ± 10.03**	23.04 ± 5.66*
ORL	12.5 ± 2.73	13.63 ± 1.60	10.75 ± 1.28	14.14 ± 3.40	35.48 ± 8.70*	25.52 ± 4.50

*Note:* Data are represented as the mean ± SD from 8 animals in each group.

Abbreviations: BC, blank control group; ORH, high‐dose OR group; ORL, low‐dose OR group; PC, positive control group.

* *p* < 0.05.

** *p* < 0.01 versus BC group.

### 3.6 Expression of PI3K, Akt, PTEN, and FSHR mRNA in Rat Ovaries

**FIGURE 10:**
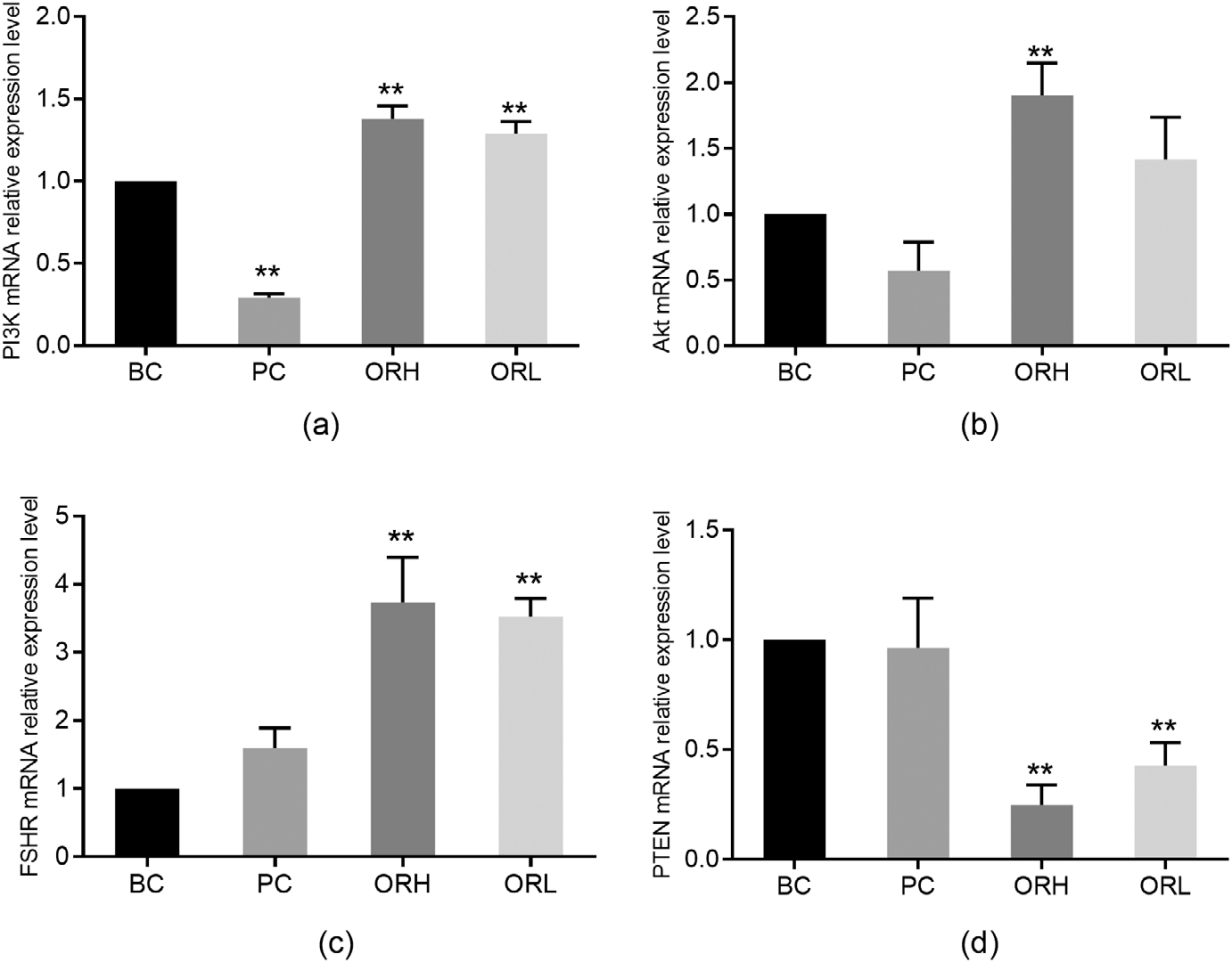
Effects of OR on mRNA expression of PI3K, Akt, FSHR, and PTEN mRNA in the ovary of rats. (a–d) shows the relative expression levels of PI3K, Akt, FSHR, and PTEN mRNA in the ovaries of each group normalized to β‐Actin mRNA expression in the BC group. BC, blank control group; ORH, high‐dose OR group; ORL, low‐dose OR group; PC, positive control group. Data are represented as the mean ± SD from 3 animals in each group. **p < 0.01 versus BC group.

Compared to the BC group, the PC group showed a significant decrease in the expression level of PI3K mRNA (*p* < 0.01). The ORH group exhibited a significant increase in the expression levels of PI3K, Akt, and FSHR mRNA (*p* < 0.01) and a significant decrease in the expression level of PTEN mRNA (*p* < 0.01). The ORL group showed a significant improvement in the expression levels of PI3K and FSHR mRNA (*p* < 0.01) and a significant reduction in the expression level of PTEN mRNA (*p* < 0.01) (Figure 10).

### 3.7 Expression of PI3K, Akt, p‐Akt, and FSHR Proteins in Rat Ovaries

**FIGURE 11:**
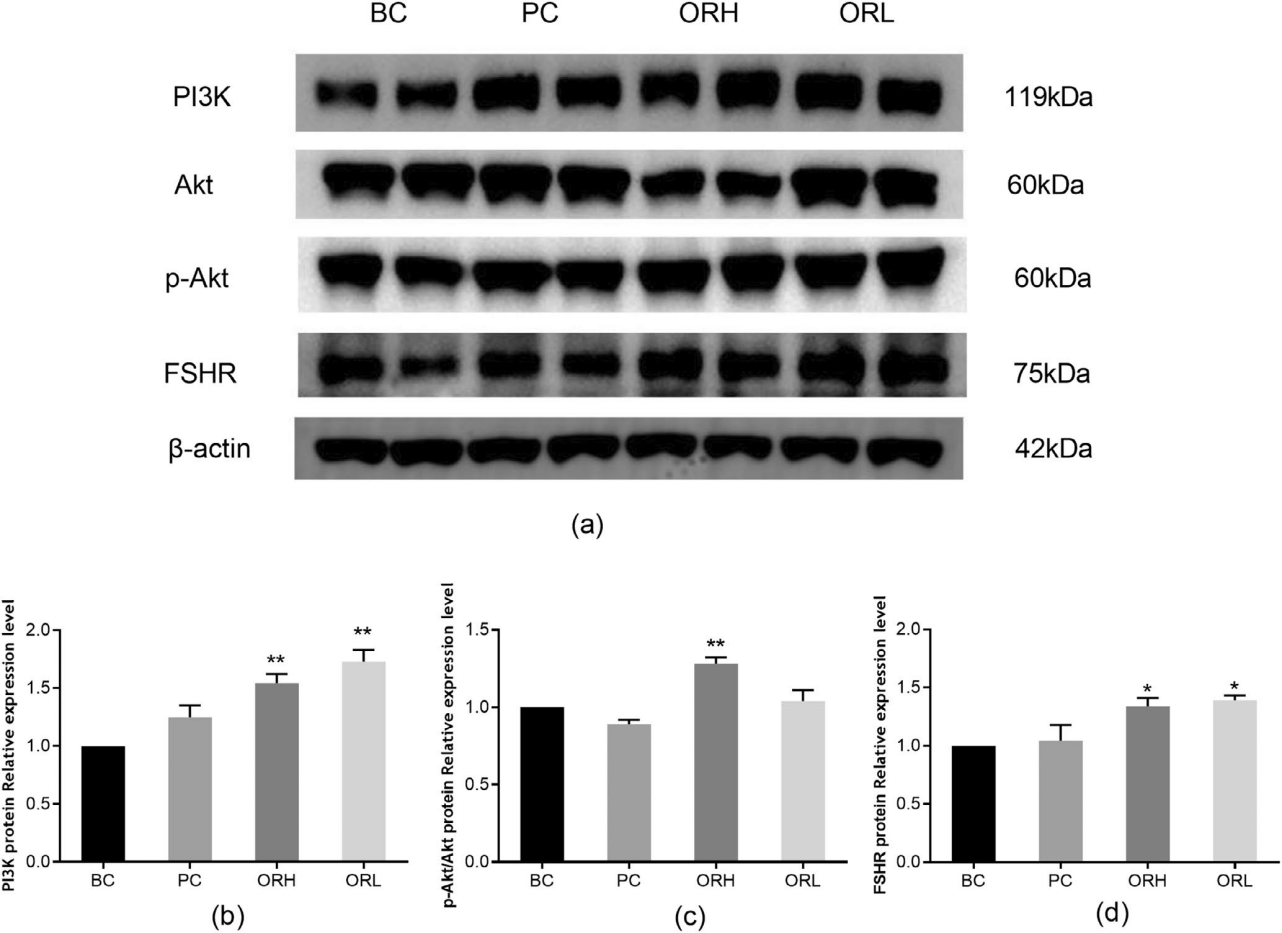
Effects of OR on protein expression of PI3K, Akt, p‐Akt, and FSHR proteins in the ovary of rats. (a) indicated representative immunoblots of PI3K, Akt, p‐Akt, and FSHR proteins in the ovaries of each group. (b) shows the relative expression levels of PI3K protein normalized to β‐Actin protein. (c) shows the phosphorylation levels of Akt protein in the ovaries of each group. (d) shows the relative expression levels of FSHR protein normalized to β‐Actin protein. BC, blank control group; ORH, high‐dose OR group; ORL, low‐dose OR group; PC, positive control group. Data are represented as the mean ± SD from 3 animals in each group. *p < 0.05 and **p < 0.01 versus BC group.

Compared to the BC group, the ORH group showed a significant increase in the expression levels of PI3K protein (*p* < 0.01), FSHR protein (*p* < 0.05), and Akt protein phosphorylation levels (*p* < 0.01). The ORL group exhibited a significant increase in the expression levels of PI3K protein (*p* < 0.01) and FSHR protein (*p* < 0.05) (Figure 11).

## 4 Discussion

This investigation delved into the effects of OR on both in vivo and in vitro follicular development. The findings elucidated that medicated serum infused with OR expedites the growth velocity of preantral follicles cultured in vitro, amplifies the secretion of estradiol from these follicles, and elevates the relative expression levels of PI3K, Akt, and FSHR mRNA. Oral administration of OR in female rats resulted in elevated serum levels of estradiol and progesterone, a notable augmentation in the count of antral follicles within the ovaries, and a reduction in both the atresia rate of antral follicles and the overall follicular atresia rate. Moreover, there was an upsurge in the expression levels of PI3K, Akt, and FSHR mRNA, a significant decrement in PTEN mRNA expression, and an enhancement in both the expression level of PI3K protein and the phosphorylation level of Akt, alongside a conspicuous elevation in FSHR protein expression. The research results verified our hypothesis that taking OR can upregulate the PI3K‐Akt signaling pathway of the ovarian follicles, thereby accelerating the growth of the follicles and reducing the follicle atresia rate. The upregulation of FSHR levels in follicles will also make the follicles more responsive to serum FSH during growth. This leads to an increase in the number and size of antral follicles in the ovaries. Not all of the increased antral follicles grow into mature follicles. Most of them will eventually become interstitial glands, which can also secrete a large amount of progesterone. These increased antral follicles and interstitial glands can secrete more estrogen and progesterone, leading to an increase in serum estrogen levels, reflecting an “estrogen‐like effect.”

In assessing the impact of OR on the growth of preantral follicles cultured in vitro, preantral follicles were isolated from the ovaries of 15‐day‐old juvenile rats. Due to ontogenetic differences between rats and humans concerning ovarian development, whereby humans exhibit follicles at various stages of development within the ovaries prenatally, whereas rats manifest primordial follicles postnatally with most follicles developing in a synchronized temporal rhythm as postnatal age progresses, it is feasible to select follicles at specific developmental stages based on the postnatal age of the rats (Rimon‐Dahari et al. 2016). In this study, the follicles isolated from 15‐day‐old rats predominantly consisted of preantral follicles with diameters ranging from 100 to 120 μm, observable under the microscope were the oocytes encased in a zona pellucida, enveloped by multiple layers of granulosa cells and a basement membrane, which upon trypan blue staining (Wycherley et al. 2004), confirmed the integrity of the basement membrane, exhibiting high survival rates the following day, robust growth vitality, capable of continuous growth for 7 days within the culture environment, thus possessing the requisite conditions to evaluate the promotive effects of OR‐medicated serum on follicular growth.

In terms of follicle isolation methods, mechanical and enzymatic approaches are mainstream. The mechanical method results in a higher survival rate of isolated follicles but demands precise handling to maintain sterility during the separation process. Conversely, the enzymatic method offers simpler operation; however, prolonged enzymatic treatment of the ovaries can lead to decreased survival and vitality of the follicles (Park et al. 2005). In this study, a combined mechanical‐enzymatic approach was adopted, involving pre‐cutting of the ovarian cortex to reduce the duration of enzymatic solution treatment on the ovaries. This not only enriches the yield of follicles but also enhances their survival rate and growth vitality. The culture medium for in vitro culture of preantral follicles is supplemented with FSH concentrations ranging from 10 to 100 mU/mL (Tanaka et al. 2019; Fujibe et al. 2019), and in the absence of FSH, follicles fail to form an antral cavity (Kumar et al. 1997; Dierich et al. 1998). Preliminary experiments observed that compared to preantral follicles cultured in a medium with 50 mU/mL FSH, those cultured in 100 mU/mL FSH yielded a higher 7‐day survival rate and greater estradiol secretion capacity. Moreover, higher FSH concentrations in the culture medium could minimize the variation in rat serum FSH levels among different groups, thereby reducing this confounding factor. Hence, the FSH concentration in all culture media was set at 100 mU/mL in this study. Medicinal serum is a common mode of drug delivery in in vitro experiments involving traditional Chinese medicine (Yin et al. 2013; Jiang et al. 2014; Li et al. 2020). The complex and diversified active components and mechanisms of action of traditional Chinese medicines, which may require metabolic processing by intestinal microbiota or transformation by liver enzymes before acting on target organs. Thus, utilizing medicinal serum as a drug delivery method is highly suitable for in vitro functional studies of traditional Chinese medicine. OR is often used in this delivery method in vitro (Ling et al. 2017). Preantral follicles cultured in a medium containing OR medicinal serum demonstrated remarkable increases in growth rate and estradiol secretion, indicating a pronounced promotive effect of OR on the in vitro growth of follicles.

Investigating the influence of OR on in vivo follicular growth, it was observed that both exogenous hormone therapy and OR intake for 14 days significantly elevate serum estradiol and progesterone levels. However, exogenous hormone therapy led to reduced FSH and LH levels due to hypothalamic feedback inhibition and was associated with decreased body, ovarian, and uterine weights, possibly related to daily medroxyprogesterone acetate intake affecting the uterine lining. In contrast, rats administered OR did not exhibit these effects and showed trends of increased body, ovarian, and uterine weights. Additionally, an increase in the number of antral follicles and a decrease in their atresia rate were observed, highlighting the nuanced “estrogen‐like effects” of OR beyond simple exogenous hormone replacement.

In preliminary transcriptomic research, our group discovered that rats administered OR orally exhibited significant differential gene expression concentrated in the PI3K‐Akt signaling pathway within their ovaries compared to control rats (He et al. 2023). This signaling pathway plays an important role in follicle growth and development (Grosbois and Demeestere 2018; Mirza‐Aghazadeh‐Attari et al. 2020; Zhou et al. 2020). PI3K transforms PIP2 into PIP3, which binds to Akt's PH domain, causing Akt to move from the cytoplasm to the cell membrane where it becomes fully phosphorylated, initiating a cascade of signaling pathways (Grosbois and Demeestere 2018). PTEN converts PIP3 back to PIP2, inhibiting the PI3K‐Akt pathway and decreasing PIP3 levels (Mirza‐Aghazadeh‐Attari et al. 2020). FSHR is expressed on granulosa and theca cells of primary follicles, with its expression increasing as the follicle develops and decreasing in atretic follicles (Magoffin 2005; Xu et al. 1995). Although there is debate over the diminishing FSHR quantity on follicles during antral development being negatively correlated with serum FSH levels (Uemura et al. 1994), it is shown that FSH in culture medium maintains FSHR expression in a dose‐dependent manner during in vitro follicle growth (Kolibianakis et al. 2009). This study confirms that OR‐medicated serum increases PI3K, Akt, and FSHR mRNA expression levels in cultured follicles; oral administration in female rats enhances ovarian PI3K, Akt, and FSHR mRNA levels, reduces PTEN mRNA levels, and elevates PI3K protein and Akt phosphorylation levels, suggesting OR promotes follicular growth by upregulating the PI3K‐Akt pathway, thereby increasing estrogen secretion through a reduction in antral follicle atresia rates and an increase in antral follicle numbers, manifesting “estrogen‐like effects”.

This study primarily investigates the effects of OR on follicular development, yet it does not focus extensively on primordial and primary follicles. This is due to the consideration that the recruitment of primordial follicles and the growth cycle of primary follicles are relatively long and independent processes, which are not easily influenced by exogenous substances. Moreover, their estrogen secretion capability is weak, which does not lead to significant fluctuations in bodily estrogen levels. Therefore, in exploring the “estrogen‐like effects” mechanism of OR, emphasis was placed on the impact of OR on the growth of late preantral follicles or early antral follicles. Consequently, in in vivo experiments, the follicle count study did not enumerate primordial follicles within the ovarian cortex, nor did it separately tally primary and secondary follicles, but instead collectively classified them as preantral follicles, focusing on the stages from preantral follicles through antral follicles to corpus luteum; in vitro experiments similarly selected late preantral follicles with a diameter of approximately 120 μm as study samples, to investigate the effects of OR‐medicated serum on the in vitro cultured growth process from preantral to antral follicles.

Our study attempts to elucidate the “estrogen‐like effects” of OR, focusing on its impact on rat follicular development both in vitro and in vivo. However, this approach presents limitations due to significant differences in the follicular recruitment and growth cycles between humans and rats. Human follicular cycles are 4–5 times longer than those of rats, and the levels of serum estradiol in humans, which fluctuate throughout the menstrual cycle, are much higher than in rats (Gougeon 1996). Additionally, unlike rats, which can ovulate multiple eggs per estrous cycle, humans typically develop only one dominant follicle per cycle, with the remaining antral follicles undergoing atresia. These differences suggest that the mechanisms underlying the “estrogen‐like effects” of OR observed in rat models may not fully translate to humans, indicating a need for further research using primates, which share more similarities with human follicular development, to fully understand the effects of OR.

## 5 Conclusion

This investigation delved into the effects of OR on both in vivo and in vitro follicular development. The utilization of OR‐medicated serum enhances the growth rate and E2 secretion capability of follicles cultured in vitro. Oral administration of OR in rats increases serum levels of estradiol and progesterone, boosts the number of antral follicles in the ovaries, and reduces follicle atresia rates, which is different from the results of taking exogenous hormones. Both in vitro and in vivo experiments demonstrate that OR can upregulate the expression of the PI3K‐Akt signaling pathway and FSHR in follicles. It is suggested that OR may promote follicular growth and development within the ovaries by modulating the PI3K‐Akt signaling pathway, thereby exhibiting “estrogen‐like effects”. This result basically verifies our hypothesis. In the next step of research, we will use the activity of OR on follicle growth and development to try to apply it to premature ovarian failure or polycystic ovary syndrome, and explore the material basis of this activity in OR.

## Author Contributions

**Hongyu Zhao:** conceptualization (lead), data curation (equal), funding acquisition (equal), methodology (lead), project administration (equal), resources (equal), writing – original draft (lead). **Yu Wang:** conceptualization (equal), data curation (equal), formal analysis (equal), methodology (equal), project administration (equal), supervision (equal), writing – original draft (supporting), writing – review and editing (equal). **Xueyuan Sun:** data curation (supporting), investigation (equal), software (equal). **Xingyao He:** data curation (equal), investigation (equal), software (equal). **Lin Di:** data curation (equal), resources (equal), supervision (equal). **Zhen Yang:** funding acquisition (equal), project administration (equal), resources (equal), supervision (lead), writing – review and editing (equal).

## Ethics Statement

All experiments were approved by the Experimental Animal Ethics Committee of the Jilin Provincial Academy of Traditional Chinese Medicine, in accordance with the committee's regulations (Approval No. JLSZKYDWLL‐2021‐019).

## Conflicts of Interest

The authors declare no conflicts of interest.

## Data Availability

The datasets used and/or analyzed during the current study are available from the corresponding author on reasonable request.
